# Sequencing, *de novo* assembly and annotation of a pink bollworm larval midgut transcriptome

**DOI:** 10.1186/s13742-016-0130-9

**Published:** 2016-06-22

**Authors:** Erica E. Tassone, Gina Zastrow-Hayes, John Mathis, Mark E. Nelson, Gusui Wu, J. Lindsey Flexner, Yves Carrière, Bruce E. Tabashnik, Jeffrey A. Fabrick

**Affiliations:** Plant Physiology and Genetics Research Unit, US Arid Land Agricultural Research Center, USDA Agricultural Research Service, Maricopa, AZ 85138 USA; DuPont Pioneer, Johnston, IA 50131 USA; DuPont Crop Protection, Stine-Haskell Research Center, Newark, DE 19711 USA; Department of Entomology, University of Arizona, Tucson, AZ 85721 USA; Pest Management and Biocontrol Research Unit, US Arid Land Agricultural Research Center, USDA Agricultural Research Service, Maricopa, AZ 85138 USA

**Keywords:** *Pectinophora gossypiella*, Pink bollworm, RNA-Seq, Transcriptome, Midgut, *Bacillus thuringiensis*

## Abstract

**Background:**

The pink bollworm *Pectinophora gossypiella* (Saunders) (Lepidoptera: Gelechiidae) is one of the world’s most important pests of cotton. Insecticide sprays and transgenic cotton producing toxins of the bacterium *Bacillus thuringiensis* (Bt) are currently used to manage this pest. Bt toxins kill susceptible insects by specifically binding to and destroying midgut cells, but they are not toxic to most other organisms. Pink bollworm is useful as a model for understanding insect responses to Bt toxins, yet advances in understanding at the molecular level have been limited because basic genomic information is lacking for this cosmopolitan pest. Here, we have sequenced, *de novo* assembled and annotated a comprehensive larval midgut transcriptome from a susceptible strain of pink bollworm.

**Findings:**

A *de novo* transcriptome assembly for the midgut of *P. gossypiella* was generated containing 46,458 transcripts (average length of 770 bp) derived from 39,874 unigenes. The size of the transcriptome is similar to published midgut transcriptomes of other Lepidoptera and includes up to 91 % annotated contigs. The dataset is publicly available in NCBI and GigaDB as a resource for researchers.

**Conclusions:**

Foundational knowledge of protein-coding genes from the pink bollworm midgut is critical for understanding how this important insect pest functions. The transcriptome data presented here represent the first large-scale molecular resource for this species, and may be used for deciphering relevant midgut proteins critical for xenobiotic detoxification, nutrient digestion and allocation, as well as for the discovery of protein receptors important for Bt intoxication.

**Electronic supplementary material:**

The online version of this article (doi:10.1186/s13742-016-0130-9) contains supplementary material, which is available to authorized users.

## Data description

### Background

The pink bollworm *Pectinophora gossypiella* (Saunders) (Lepidoptera: Gelechiidae) is an important global pest of cotton. In many countries, transgenic cotton producing *Bacillus thuringiensis* (Bt) crystalline (Cry) proteins kills pests including the pink bollworm, thereby providing economic and environmental benefits. However, the evolution of pest resistance threatens the continued success of such Bt crops. While field populations of the pink bollworm in the USA have remained susceptible to two different Cry toxins produced simultaneously in Bt cotton, field-evolved practical resistance to Bt cotton has occurred widely in India [1–3].

Cry toxins kill susceptible pests like the pink bollworm by binding to protein receptors on the surface of midgut epithelial cells, eventually causing cell lysis [4]. In this study, we used Illumina sequencing of cDNA from the larval midgut of a Bt-susceptible strain to provide the first comprehensive view of the genes transcribed in this species. We generated over 18 million high-quality DNA sequence reads and >35 million bases that assembled into 21,715 unique transcripts. This transcriptome sequencing effort has dramatically increased the number of known genes for this insect, and provides an invaluable resource for the discovery of potential roles of proteins involved in various physiological and toxicological processes in the pink bollworm larval midgut.

### Samples

Samples were derived from the APHIS-S strain of pink bollworm maintained at the US Department of Agriculture (USDA) Agricultural Research Service US Arid Land Agricultural Research Center in Maricopa, Arizona. APHIS-S is a Bt-susceptible strain that has been reared in the laboratory for more than 30 years without exposure to Bt toxins [5]. To generate samples, 120 newly emerged neonates were placed individually on ~5 g of wheat germ pink bollworm diet in 30 ml plastic cups and reared at 26 °C with ~30 % relative humidity and a photoperiod of 14 light:10 dark. After 9 days, midguts were dissected from three sets of ten female and ten male 4^th^ instar larvae. Salivary glands, foregut, Malpighian tubules and hindguts were removed from each midgut in phosphate buffered saline buffer. Three biological replicates of 20 midguts each were pooled in 0.5 ml RNAlater (Sigma, St. Louis, USA), held overnight at 4 °C and stored at −80 °C. Frozen midguts in RNAlater were shipped to DuPont Pioneer in Johnston, Iowa, USA for RNA extraction, library preparation and DNA sequencing.

### Sequencing

Total RNAs were isolated from frozen midgut pools using the Qiagen RNeasy kit (Hilden, Germany). Sequencing libraries from the resulting total RNAs were prepared using the TruSeq mRNA-Seq kit and protocol from Illumina, Inc. (San Diego, USA). Briefly, mRNAs were isolated via attachment to oligo(dT) beads, chemically fragmented and then reverse transcribed into cDNA via random hexamer priming. Resulting double stranded cDNA fragments were end-repaired to create blunt end fragments, 3′ A-tailed, ligated with Illumina indexed TruSeq adapters and PCR amplified using Illumina TruSeq primers. Purified PCR amplified libraries were assessed for quality and quantity on the Agilent Bioanalyzer DNA 7500 chip before normalization and sample pooling.

Sample pools were clustered and sequenced on the Illumina HiSeq 2500 system with Illumina TruSeq SBS Rapid v1 chemistry as per vendor protocols. Samples selected for transcriptome assembly were paired-end sequenced, with 76 cycles per read to a target depth of 40 million read pairs per sample. Raw quality was assessed and filtered with a custom pipeline that uses both the program FastQC and Trimmomatic (V 0.32), using the parameters ILLUMINACLIP:TruSeq3-PE.fa:2:30:10 LEADING:10 TRAILING:20 SLIDINGWINDOW:4:25 MINLEN:36 to remove adaptor sequence and filter by quality score. After filtering, approximately 18 million reads were obtained, totaling over 5 Gb or 2 × 72 bp paired-end data. The short read archive (SRA) accessions for data used in the assembly are as follows: SRX1164974, SRX1164977 and SRX1164978.

### Transcriptome assembly

Before assembly, the three datasets were concatenated and read abundance was normalized to 50× coverage, using the *in silico* normalization tool in Trinity to improve assembly time and minimize memory requirements. Filtering and normalization reduced the dataset to 3 Gb, comprised of approximately 9 million read pairs that were then assembled using default parameters in Trinity (v.2.0.6) with the addition of the ‘-- jaccard clip’ flag to reduce the generation of transcript fusions from non-strand-specific data. Transcript expression levels were estimated with RSEM [6] and open reading frames were predicted using Transdecoder [7]. To remove bacterial contamination from the assembly, a BLASTx analysis of the newly assembled transcriptome was performed against a custom bacterial database containing all bacterial sequences deposited in NCBI (created 18 August 2015). After contamination filtration, the transcriptome was again filtered, sorted and prepared for NCBI transcriptome shotgun assembly (TSA) submission as previously described [8]. The resulting transcriptome was analyzed using TransRate (v.1.0.1), obtaining a TransRate score of 0.21, which indicates that the assembly is better than ~50 % of 155 published *de novo* transcriptomes available in the NCBI TSA [9].

### Annotation

Functional annotation was performed at the peptide level using a custom pipeline [8] that defines protein products and assigns transcript names. Predicted proteins/peptides were analyzed using InterProScan5, which searched all available databases including Gene Ontology (GO) [10]. BLASTp analysis of the resulting proteins was performed with the UniProt Swiss-Prot database (downloaded 11 February 2015). Annie [11], a program that cross-references Swiss-Prot BLAST and InterProScan5 results to extract qualified gene names and products, was used to generate the transcript annotation file. The resulting .gff3 and .tbl files were further annotated with functional descriptors in Transvestigator [12].

### Transcriptome comparisons

The assembled pink bollworm transcriptome was compared with midgut transcriptomes from three other lepidopterans, *Plutella xylostella* [13], *Chilo suppressalis* [14] and *Heliothis virescens* [15], and was found to have comparable metrics (Table 1). Specifically, the number of assembled contigs per 1000 reads was 2.5 for pink bollworm, 0.94 for *C. suppressalis*, 0.30 for *H. virescens* and 5.4 for *P. xylostella*. The number of BLASTx sequence hits (cutoff e-value of 10^−5^) in the non-redundant (nr) NCBI protein database per 1000 assembled reads was 1.2 for pink bollworm, 0.39 for *C. suppressalis*, 0.14 for *H. virescens* and 0.72 for *P. xylostella* (Table 1).

**Table 1:** Comparison of assembled lepidopteran transcriptomes

	*Chilo suppressalis*	*Heliothis virescens*	*Plutella xylostella*	*Pectinophora gossypiella*
Platform	Illumina	Illumina, Roche, 454, Sanger	Illumina	Illumina
Assembled reads	39,400,002	212,987,028	39,764,230	18,623,508
Average read size (bp)	90	Variable	90	72
Number of contigs	37,040	63,648	213,674	46,458
Contigs per 1000 reads	0.94	0.30	5.4	2.5
Mean contig size (bp), range	497, 201–9744	383, 80–2000	189, nr	770, 224–14,619
Sequences with e-value <10^−5^	15,446	29,978	28,768	21,715
Number of e-value <10^−5^ hits Per 1000 reads	0.39	0.14	0.72	1.2
GC (%)	42	nr^a^	nr	39
N_50_ transcript length (bp)	nr	1031	262	1153
Pipeline	Trinity	SeqMan Ngen v2.1	Trinity	Trinity
Reference	[14]	[15]	[13]	This study

^a^ *nr* not reported

The quality of the pink bollworm assembly was further assessed by direct comparison of core statistics with the *P. xylostella* midgut transcriptome [13] (Table 2). We evaluated the completeness of both the *P. xylostella* and *P. gossypiella* transcripts using the program BUSCO (benchmarking universal single-copy orthologs) using the arthropod gene set [16]. The percentages of conserved genes from the *P. gossypiella* and *P. xylostella* transcriptomes recovered by the BUSCO analysis are ~34 and ~37 %, respectively. The overall BUSCO percentages are lower than previously reported for a reference *Spodoptera frugiperda* transcriptome [17], but are not surprising given these are single-organ (i.e., midgut) assemblies compared with assembled RNA sequences from whole larvae and tissue samples from multiple time points. Lastly, a tBLASTx analysis of the *P. gossypiella* transcriptome against the *P. xylostella* midgut transcriptome (representing the nearest phylogenetic relative lepidopteran relative with a currently available midgut transcriptome) revealed that 29 % (12,475 out of 46,458), 37 % (17,032 out of 46,458) and 91 % (42,089 out of 46,458) had matching hits at e-values of 10^−5^, 10^−2^ and 10^1^ [18]. These results are not unexpected for tBLASTx at the more stringent e-values given the considerable phylogenetic distance between the two species of Lepidoptera [19].

**Table 2:** Comparison of *Pectinophora gossypiella* and *Plutella xylostella* midgut transcriptomes by BUSCO analysis^a^

Species	Complete (%)	Duplicated (%)	Fragment (%)	Missing (%)
*P. gossypiella*	34	8.8	30	35
*P. xylostella*	37	12	24	38

^a^A total of 2675 total BUSCO groups were searched from the assembled *P. gossypiella* midgut transcriptome and the assembled *P. xylostella* midgut short read archive transcriptome

### Gene ontology

**Fig. 1:**
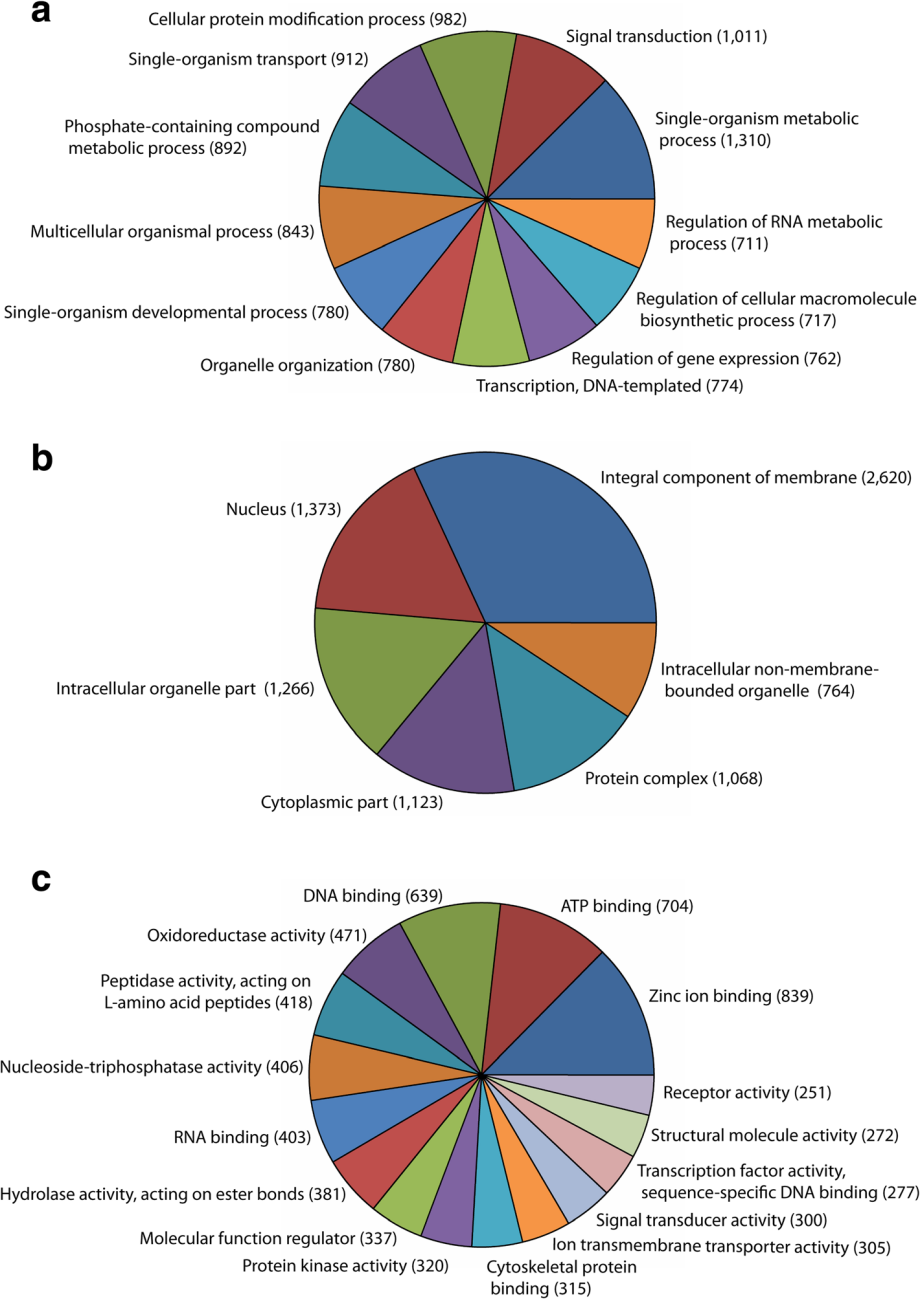
Classification of *Pectinophora gossypiella* midgut transcripts based on predicted Gene Ontology (GO) terms. **a** Biological Process, (**b**) Cellular Component and (**c**) Molecular Function GO terms were determined using Blast2Go [19, 20] with an e-value cutoff of 10^−3^, and minimum sequence filters set at 707 sequences for Biological Process, 640 for Cellular Component and 250 for Molecular Function for generating pie charts. Note that individual categories can have multiple mappings, resulting in a sum greater than the total number of transcript sequences assigned GO terms

Blast2GO [20, 21] was used to assign *P. gossypiella* transcripts with a minimum BLASTx e-value of 10^−3^ into putative functional groups or GO terms. A total of 12,762 transcript sequences were assigned GO terms (Additional file 1, [18]), including 7073 with hits at the Biological Process level, 6402 at the Cellular Component level and 7747 sequences at the Molecular Function level. Within the Biological Process GO category, the most abundant transcripts were assigned to ‘single-organism metabolic process’, ‘signal transduction’ and ‘cellular protein modification’ (Fig. 1a). ‘Integral component of membrane’, ‘nucleus’ and ‘intracellular organelle part’ were the most abundant GO terms for Cellular Component (Fig. 1b). For Molecular Function, ‘zinc ion binding’, ‘ATP binding’ and ‘DNA binding’ were the most prevalent, with several different types of hydrolases also highly represented (Fig. 1c). Overall, typical gut-specific functions, such as digestion and storage, energy metabolism, ion transport and gene regulation were indicated by GO terms (Additional file 1, [18]).

## Abbreviations

Bt, *Bacillus thuringiensis*; BUSCO, benchmarking universal single-copy orthologs; Cry protein, crystalline protein; GO, gene ontology; NCBI, National Center for Biotechnology Information; nr, non-redundant; SRA, short read archive; TSA, transcriptome shotgun assembly; USDA, US Department of Agriculture

## Additional file

Additional file 1: Bollworm_GO_26APR16.csv. CSV file of gene ontology terms for pink bollworm transcripts. (CSV 2338 kb)
